# Improved CRISPR/Cas9 gene editing in primary human myoblasts using low confluency cultures on Matrigel

**DOI:** 10.1186/s13395-021-00278-1

**Published:** 2021-09-22

**Authors:** Hayley Goullée, Rhonda L. Taylor, Alistair R. R. Forrest, Nigel G. Laing, Gianina Ravenscroft, Joshua S. Clayton

**Affiliations:** 1 Centre for Medical Research, Faculty of Health and Medical Sciences, The University of Western Australia, Nedlands, WA Australia; 2 Harry Perkins Institute of Medical Research, 6 Verdun St, Nedlands, WA 6009 Australia; 3 School of Biomedical Science, Faculty of Health and Medical Sciences, The University of Western Australia, Nedlands, WA Australia

**Keywords:** CRISPR, Gene editing efficiency, Primary human myoblasts, Matrigel, Confluency

## Abstract

**Background:**

CRISPR/Cas9 is an invaluable tool for studying cell biology and the development of molecular therapies. However, delivery of CRISPR/Cas9 components into some cell types remains a major hurdle. Primary human myoblasts are a valuable cell model for muscle studies, but are notoriously difficult to transfect. There are currently no commercial lipofection protocols tailored for primary myoblasts, and most generic guidelines simply recommend transfecting healthy cells at high confluency. This study aimed to maximize CRISPR/Cas9 transfection and editing in primary human myoblasts.

**Methods:**

Since increased cell proliferation is associated with increased transfection efficiency, we investigated two factors known to influence myoblast proliferation: cell confluency, and a basement membrane matrix, Matrigel. CRISPR/Cas9 editing was performed by delivering Cas9 ribonucleoprotein complexes via lipofection into primary human myoblasts, cultured in wells with or without a Matrigel coating, at low (~ 40%) or high (~ 80%) confluency.

**Results:**

Cells transfected at low confluency on Matrigel-coated wells had the highest levels of transfection, and were most effectively edited across three different target loci, achieving a maximum editing efficiency of 93.8%. On average, editing under these conditions was >4-fold higher compared to commercial recommendations (high confluency, uncoated wells).

**Conclusion:**

This study presents a simple, effective and economical method of maximizing CRISPR/Cas9-mediated gene editing in primary human myoblasts. This protocol could be a valuable tool for improving the genetic manipulation of cultured human skeletal muscle cells, and potentially be adapted for use in other cell types.

**Supplementary Information:**

The online version contains supplementary material available at 10.1186/s13395-021-00278-1.

## Introduction

CRISPR/Cas9 is now an essential tool in the field of genomics. First described in 2012 [1], this system uses a programmable guide RNA (gRNA) to target the Cas9 endonuclease to a specific DNA sequence for cleavage. Such precision editing of the genome has accelerated the development of disease models, genetic therapies, and our understanding of the genome as a whole [1–5].

Cas9 can be delivered to cells as DNA (via plasmid), mRNA or protein. The latter method, first described in 2014 by Kim et al. [6], involves the delivery of a purified Cas9 enzyme pre-complexed with the gRNA to form a Cas9/gRNA ribonucleoprotein (RNP) complex. This method has since become favored in many contexts due to higher editing efficiency and reduced off-target effects compared to plasmid-based or mRNA delivery [6–9]. Although CRISPR/Cas9 is a relatively simple and flexible system, efficient delivery of the components into some cell types remains a major challenge [8, 10, 11]. The most commonly used transfection method is lipid-based (lipofection), due to its ease of use and economy [12]. However, there is no one-size-fits-all protocol for different cell types. Some cells are highly amenable to transfection, while others are refractory and/or have a high rate of mortality following transfection [13].

In the context of neuromuscular disorders, cultured myoblasts are a valuable tool for studying cell biology and disease mechanisms. Two of the most commonly used in vitro muscle models are immortalized C2C12 myoblasts and stem cell-derived muscle cultures [14–18]. However, C2C12s are derived from mice [19] and thus have differing myogenic mechanisms to humans—for example, knockdown of *MYOD1* does not affect *MYF5* expression in human myoblasts, whereas Myf5 protein production is increased in *Myod1*-null murine myoblasts [20, 21]. While an effective model, a human genomic context is particularly pertinent for genome editing studies. Additionally, C2C12s are often genomically unstable with abnormal karyotypes [22–24]. Stem cells are similarly prone to mutagenesis and aneuploidy due to frequently defective DNA repair mechanisms [25–28]. While both C2C12s and stem cells are invaluable tools for muscle research, we wanted to expand the arsenal of skeletal muscle models that (i) maintain stable gene expression profiles in the human genomic context and (ii) can be efficiently transfected and/or edited for molecular studies. Thus, we focused on improving transfection and editing efficiency of primary human myoblasts.

While often considered a better reflection of the in vivo environment, the nature of primary cells presents many practical limitations. Unlike established cell lines, they have a limited lifespan and cannot undergo serial passaging [29]. Primary myoblasts in particular are refractory to transfection, especially compared to immortalized myoblasts such as C2C12s [30]. Transfection of myoblasts have traditionally been carried out via delivery of DNA plasmids—here, lipofection efficiency is regularly < 10%, and rarely surpasses 50% [31–33]. Muscle-based studies that have used RNP cargo have primarily been performed on stem cells prior to myogenic differentiation, and/or use other methods of delivery [34, 35]. Many studies have attempted to improve myoblast transfection efficiency using various transfection methods and cell selection processes [30, 32, 33, 36–42]. For example, electroporation and nucleofection enable higher transfection efficiencies compared to lipofection. However, these methods are expensive by comparison, requiring specialized equipment and much higher quantities of CRISPR/Cas9 reagents [43–45]. Fluorescence-activated cell sorting (FACS) can be used to enrich for cells containing fluorescently-tagged CRISPR/Cas9 components [46, 47]. Edited cells can also be single-cell cloned to generate homogenous (clonal) cell populations that contain the desired edit [48]. However, primary myoblasts lose their differentiation potential following single-cell cloning, and those that have been successfully edited often do not survive the stress of sorting (our own unpublished findings). Without the option of producing clonal populations, obtaining reliable and informative data from bulk populations requires as high an editing efficiency as possible. Therefore, we used a systematic approach to identify factors that influence transfection and editing efficiencies in primary myoblasts.

Cellular proliferation rate has previously been linked to transfection efficiency, where transfection success was measured based on transgene expression [13, 49]. Consequently, manipulation of factors that influence proliferation rate has the potential to improve transfection efficiency. The rate of proliferation within a myoblast culture is intrinsically linked to confluency [50, 51]. Most protocols accompanying popular transfection reagents recommend relatively high cell confluency at the time of transfection—60–90% confluency for Lipofectamine™ 2000, 3000, and RNAiMAX (Invitrogen™), Fugene® (Promega), and Alt-R® CRISPR/Cas9 RNP (Integrated DNA Technologies). However, myoblasts begin to fuse to form myotubes at ~ 70% confluency and cease to proliferate [41], thus becoming refractory to transfection. Transfection at lower cell density (e.g., during the period of greatest myoblast proliferation) is likely to improve transfection efficiency. This is supported by Jackson et al., who reported a confluency of 40% to be optimal for plasmid-based transfections of C2C12 myoblasts [36]. Another factor known to enhance myoblast proliferation is the use of extracellular matrices such as Matrigel, a formulation consisting primarily of laminin, collagen IV, and enactin [38, 52, 53]. Accordingly, Balci and Dincer showed that seeding C2C12 myoblasts on Matrigel increased their proliferation and improved lipid-mediated plasmid uptake by > 2-fold [38].

While low cell confluency and Matrigel have independently been shown to increase C2C12 myoblast transfection efficiency, no studies have investigated these strategies in combination. Understanding the relationship between Matrigel, confluency, transfection efficiency, and CRISPR/Cas9-mediated editing may help maximize editing in a bulk population of primary myoblasts. This in turn could enhance the ability to study human skeletal muscle function in response to genetic manipulation.

Here, we report an optimized transfection protocol that uses Lipofectamine™ CRISPRMAX™ to efficiently deliver CRISPR/Cas9 RNPs to three primary human myoblast lines. Contrary to most commercial protocols, CRISPRMAX™ recommends optimizing transfection across a broader range of cell densities (30–70% confluency), allowing us to test cell densities at the low and high end of the spectrum. We thus compared transfection and editing efficiency of cells transfected at low or high confluency, both with and without Matrigel. By testing these combinations, we established a protocol that enabled an average of >50%, and up to 93.8%, editing in human primary myoblasts. The workflow we describe could be used to optimize transfection and editing conditions for other hard-to-transfect cell types.

## Methods

### Human primary myoblast culture

Three primary human skeletal muscle cell lines (skMDCs) were obtained from Cook MyoSite®. All lines were derived from the rectus abdominus of healthy donors: an 18-year-old Caucasian male (18M; donor product lot #01236-18M), a 32-year-old Caucasian female (32F; donor product lot #01034-32F) and a 42-year-old Caucasian female (42F; donor product lot #01269-42F). G-banding was performed to confirm normal diploid karyotype (PathWest Diagnostic Genomics laboratory). SkMDCs were maintained at 37°C and 5% CO_2_ in MyoTonic™ Basal Media (Cook MyoSite®) and Growth Supplement (Cook MyoSite®), supplemented with 10% FBS (Gibco), 50 U/mL penicillin and 50 mg/mL streptomycin. Where required, cell culture plates were coated with Matrigel (Standard LDEV-Free Formulation, Corning®) diluted 1:100 in Dulbecco’s modified eagle medium F12 (DMEM/F12, in-house) and incubated at 37°C for 1 h. Residual media was aspirated immediately prior to use.

### Transfection of skMDCs

One day prior to transfection, cells (passage 7 or below) were seeded in Matrigel-coated (Mat^pos^) and uncoated (Mat^neg^) 12-well plates. Cells were manually counted using a haemocytometer and standard trypan blue staining. They were then plated at either the high/commercially recommended confluency (Hi, ~ 4.8 × 10^4^ cells/well; ~ 1.4 × 10^4^ cells/cm^2^) or at low confluency (Lo, ~ 2.4 × 10^4^ cells/well; ~ 6.8 × 10^3^ cells/cm^2^), using a serial dilution. Cell confluence at the time of transfection (~ 24 h after seeding) was ~ 80% (Hi) or ~ 40% (Lo).

Immediately prior to transfection, the center of each well was imaged at 4x objective magnification using a phase-contrast microscope (Olympus IX71 microscope, Olympus CellSens Standard 2.3 software). Imaging was performed on the central area of the well at all time points for all conditions. The number of cells in each image was counted using ImageJ 1.52i (ImageJ, U.S. National Institutes of Health, Bethesda, MD, USA). In brief, all images were converted to 8-bit grayscale and pixel threshold limits set to 0 and 165 to detect myoblasts. The ‘Analyze Particles’ tool was used to tally the number of cells by counting particles with an area > 200 μm^2^. The final count was scaled based on the surface area of the well.

To assess CRISPR/Cas9 editing, we targeted two well-characterized myogenic regulatory factors (*MYF5* and *MYOD1*), and a recently identified myogenic regulator; the highly conserved bone morphogenic protein (BMP) agonist, *GREM1* [54]. Alt-R® CRISPR/Cas9 tracrRNA ATTO™550 (IDT) and guide RNAs with XT modification (IDT, see Additional file 1 for sequences) were resuspended in Nuclease-Free Duplex Buffer to a concentration of 100 μM. Each guide (crRNA) was combined 1:1 with the tracrRNA, annealed by heating at 95°C for 5 min and then slowly cooling at room temperature. The resultant single guide RNA (sgRNA) complex was diluted 1:100 in Opti-MEM (Gibco™) to 1 μM. Alt-R® S.p. Cas9 Nuclease V3 (IDT) was diluted to 1 μM in Opti-MEM immediately prior to use.

CRISPR/Cas9 components were delivered to skMDCs using Lipofectamine™ CRISPRMAX™ Cas9 Transfection Reagent (Invitrogen™). Transfections were performed as described in the IDT handbook “Alt-R® CRISPR/Cas9 System: Cationic lipid delivery of CRISPR ribonucleoprotein complex into mammalian cells” [55]. Volumes were scaled up 8-fold for use in 12-well plates. An untreated control (UT, *i.e.* no RNP) and a negative control (NC, i.e., a scrambled guide) were included for each cell line and condition. Experiments were first optimized on a single cell line before being repeated on all three lines. Repeat experiments were performed for some guides and conditions to confirm findings.

Media was refreshed 24 h after transfection to remove cell debris and extracellular fluorescent complexes prior to imaging. Wells were imaged again at 48 h and harvested. Cells were rinsed with 1× Dulbecco’s phosphate buffer saline (DPBS), dissociated by incubation with 0.05% Trypsin-EDTA (in-house) for 5 min at 37 °C, and pelleted by centrifugation at 300×*g* for 5 min. The pellet was either snap-frozen on dry ice for DNA extraction or resuspended in FACS buffer for sorting.

### Fluorescence-activated cell sorting (FACS) and analysis

Total cells from each well were resuspended in 200 μL FACS buffer (25 mM 1 M HEPES, pH 7.5; 2 mM 0.5 M EDTA, pH 8; 1% FBS in DPBS) and processed on a BD FACSAria™ II using BD FACSDiva™ software (Version 6.1.3). Gates were set on untreated samples for each cell line using a 550-nm laser to detect ATTO 550 and a 640-nm laser to detect APC-Cy7 as a negative control. Data analysis was performed using FlowJo (Version 10.0.7). Median fluorescence identify (MFI) for ATTO 550 was calculated on single cells (gated based on FSC and SSC). Transfection efficiency was calculated as the percentage of ATTO 550 positive (ATTO 550^+^) cells divided by the total number of analyzed cells (*n* = 10,000 cells per sample). Thresholds for ATTO^+^ cells were set based on matched untreated samples. Following FACS, each sample was pelleted by centrifugation at 300×*g* for 5 min for DNA extraction.

### DNA extraction from skMDCs

DNA was extracted from skMDC cell pellets using the QIAamp DNA Mini Kit (QIAGEN) as per the manufacturer’s instructions, but with minor modifications. In brief, cell pellets were resuspended in 100 μL PBS and subsequent kit volumes scaled accordingly. QIAamp MinElute spin columns (QIAGEN) were used for DNA purification as these allow small elution volumes (10 μL), maximizing the concentration of the extracted DNA. DNA was eluted in 10–15 μL UltraPure™ DNase/RNase-Free Distilled Water (Invitrogen™). DNA concentration and purity were measured using a NanoDrop™ One spectrophotometer (ThermoFisher).

### Polymerase chain reaction (PCR) and Sanger sequencing

All PCR reactions were performed using GoTaq® 2× Colorless Master Mix (Promega) as per the manufacturer’s instructions with 50 ng DNA per reaction. *MYF5* reactions contained 20% Q-solution (from QIAGEN *Taq* DNA Polymerase kit). *MYOD1* reactions were supplemented with 5% DMSO. Primer sequences and thermocycling conditions are listed in Additional file 2. PCR amplicons were analyzed on a 1% agarose gel to confirm size and specificity. Amplicon purification and bidirectional Sanger sequencing were performed at the Australian Genome Research Facility, Perth.

### Quantitative polymerase chain reactions (qPCRs)

All qPCRs were performed using the Rotor-Gene SYBR® Green PCR Kit (QIAGEN), as per the manufacturer’s instructions. Reactions were carried out on a Rotor-Gene Q 5plex HRM PCR cycler and results visualized using Rotor-Gene Q Series Software, Version 2.3.1. A housekeeping gene, *EEF2,* was used for normalization. Primer sequences and thermocycling conditions are listed in Additional file 3.

### Data analysis and statistics

Editing efficiency was calculated from Sanger chromatograms using the online tool TIDE (Tracking of Indels by Decomposition) [56]. Statistical analysis was performed using GraphPad Prism 8.0.1. All reported values are the mean ± SD of at least three replicates per experiment. One-way analysis of variance (ANOVA), followed by Holm-Sidak or Tukey post hoc tests (as appropriate), were used to test for differences among groups of three or more. Two-way Repeated-Measures (RM) ANOVAs followed by Sidak’s multiple comparison tests were used to assess differences between cell counts over time. Relationships between two variables were determined using linear-regression analyses. Paired *t* tests were used to assess the difference in gene expression levels between negative controls and knockouts. The significance threshold for all statistical tests was defined as *p* < 0.05.

## Results

### Matrigel increases proliferation of skMDCs

**Fig 1:**
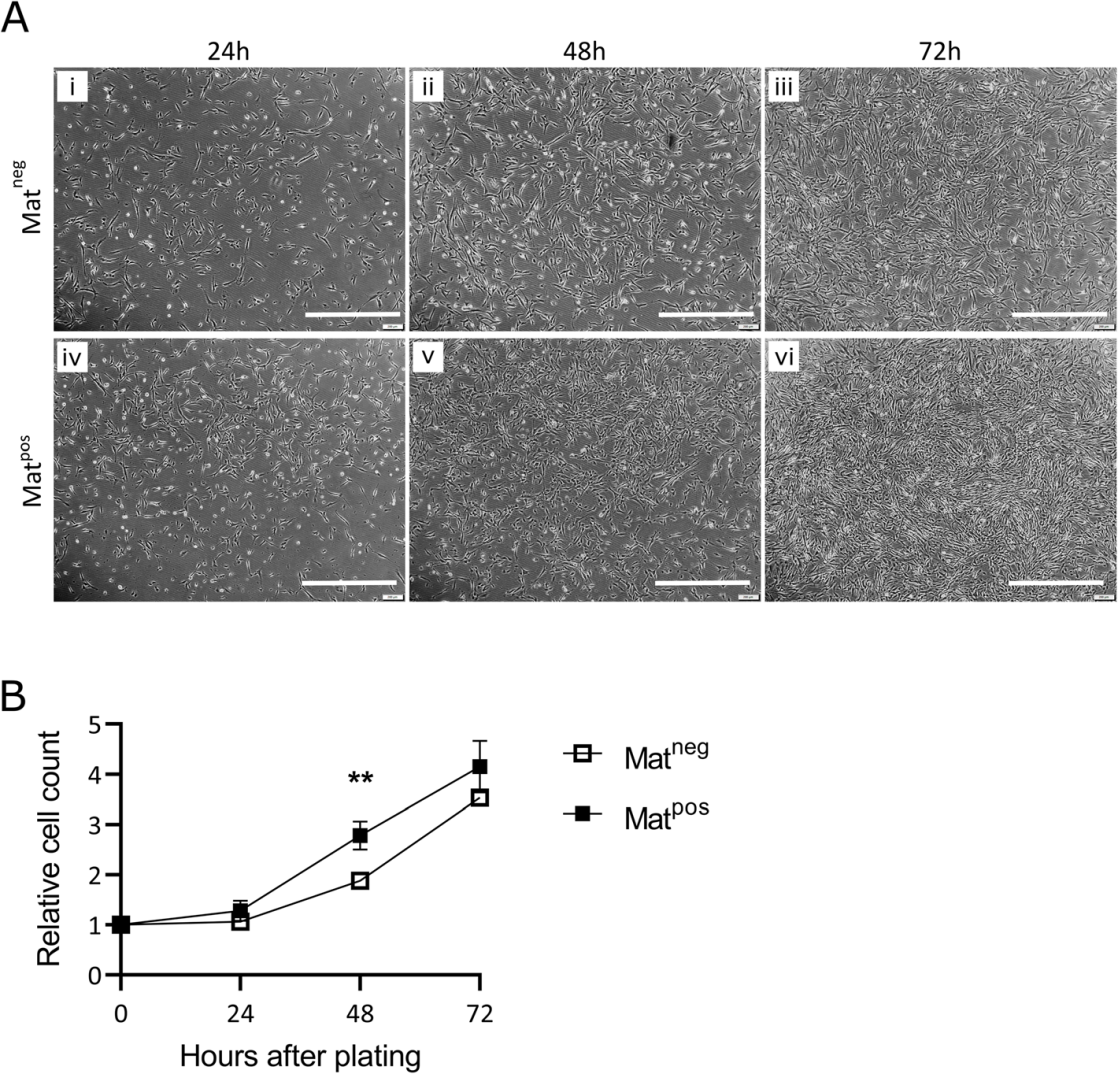
Plating on Matrigel increases proliferation of primary human myoblasts (skMDCs). **A** Representative phase contrast images of untreated cells in Matrigel coated (Mat^pos^; iv, v, vi) and uncoated (Mat^neg^ ; i, ii, iii) wells at 24, 48, and 72 h after seeding (~ 6.8 × 10^3^ cells/cm^2^). **B** Proliferation rate of three skMDC lines, relative to the number of cells plated (value = 1 at 0 h). Data are reported as mean ± SD. Statistics were performed using a two-way repeated measures ANOVA with Sidak’s multiple comparison. *n =* 5 (1× 18 M, 1× 32F, 3× 42F); ** = p < 0.01. Scale bar = 1 mm

The positive effect of Matrigel on cell proliferation is well established [38, 52, 53]. We performed a small experiment to confirm that the same effect would be seen in our skMDC lines. We achieved this by counting the number of untreated cells in Mat^pos^ and Mat^neg^ wells at 24, 48, and 72 h post-plating (Fig. 1A). At 24 h, there was no significant difference in cell number in Mat^pos^ versus Mat^neg^ conditions (*p* = 0.41) (Fig. 1B). However, by 48 h Mat^pos^ wells had ~ 1.5 times more cells than Mat^neg^ wells (*p* = 0.0067). As confluency increased proliferation slowed, and by 72 h the difference between the two conditions was no longer significant (~ 1.2 fold difference; *p* = 0.27). In support of previous reports, these data show that skMDCs seeded on Matrigel have a higher rate of proliferation compared to cells grown on uncoated wells. Further, it shows that the skMDCs follow the typical sigmoidal growth curve of mammalian cells [57], with proliferation slowing as cell numbers increase.

### Maximal transfection was achieved using Matrigel and low cell confluency

**Fig 2:**
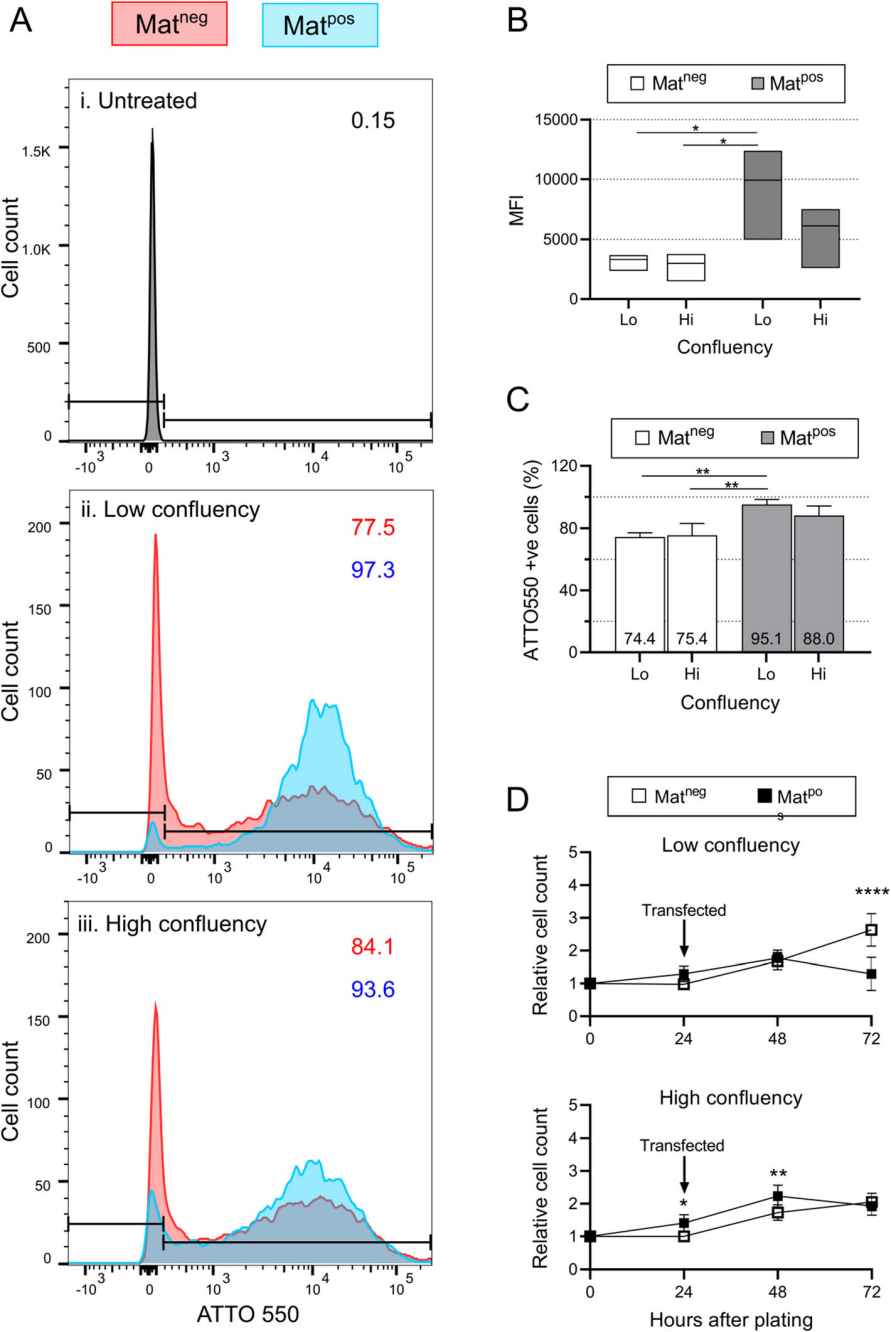
Maximal transfection of skMDCs was achieved using Matrigel and low cell confluency. Fluorescence activated cell sorting (FACS) was used to analyze the proportion of ATTO 550^+^ cells under different transfection conditions. **A** Images from skMDC-42F are shown as representative images of all three lines. ATTO 550^+^ gates were set on the untreated sample (i). The X axes show the median fluorescence intensity (MFI) for ATTO 550 in cells transfected at low (ii) and high (iii) confluency. The percentage of cells that are ATTO 550^+^ are shown for Mat^neg^ (red) and Mat^pos^ (blue) conditions. **B** The MFI is highest in cells transfected under Lo/Mat^pos^ conditions. Box plots illustrate minimum, maximum and median MFIs across the 3 cell lines. **C** Transfection efficiencies were quantified in three skMDC lines under different conditions. Data represent mean ± SD. One-way ANOVA with Holm-Sidak’s multiple comparison test was performed, n=3 per condition (1× 18M, 1× 32F, 1× 42F); ** = p < 0.01. **D** Proliferation rate of transfected skMDCs relative to the number of cells plated (values normalised to 1 at 0 h). Data reported are mean ± SD. Two-way ANOVA with Sidak’s multiple comparison was performed, n = 5 (1× 18M, 1× 32F, 3× 42F); * = p < 0.05; ** = p < 0.01; **** = p < 0.0001. UT = Untreated; Lo = low confluency, Hi = high confluency; Mat^*neg*^ *= uncoated wells; Mat*^*pos*^ *= Matrigel coated wells*

To assess the impact of confluency and Matrigel together on transfection efficiency, we measured the proportion of myoblasts that were gRNA-ATTO 550 positive following transfection under different conditions. Cells transfected under Lo/Mat^pos^ conditions had the highest median fluorescence intensities (MFIs) (Fig. 2A, B), significantly larger than both Lo and Hi/Mat^neg^ conditions (*p* = 0.039 and *p* = 0.028, respectively) but not Hi/Mat^pos^ (*p* = 0.28). This suggests that successfully transfected Lo/Mat^pos^ cells contained more RNP complexes per cell compared to those transfected under other conditions. As well as a higher rate of transfection at the single cell level, Lo/Mat^pos^ conditions transfected the greatest proportion of cells in all three skMDC donor lines (95.1% ± 2.58). This was significantly greater than both Lo/Mat^neg^ (74.4% ± 2.17, *p* = 0.0088) and Hi/Mat^neg^ conditions (75.4% ± 6.26, *p* = 0.0098), but not Hi/Mat^pos^ conditions (87.97% ± 5.07, *p* = 0.26) (Fig. 2C). There was no significant difference between Hi/Mat^pos^ and either Lo/Mat^neg^ or Hi/Mat^neg^ conditions (*p* = 0.26 and *p* = 0.061, respectively).

Previous studies have also correlated lipofection efficiency with increased toxicity in multiple cell lines [12, 13]. To assess whether this applied to skMDCs, we counted the number of cells at the time of transfection, then 24 h and 48 h post-transfection. Under both low and high confluency conditions, the number of Mat^pos^ cells begins to decline within 24 h post-transfection while the Mat^neg^ skMDCs continue on an upward trajectory (Fig. 2D). At 48 h post-transfection, the number of cells in Lo/Mat^pos^ conditions was significantly less than those under Lo/Mat^neg^ conditions (*p* < 0.0001). The same negative relationship between transfection and cell viability was seen for all 3 donor human myoblasts lines (Fig. 2D).

Overall, our results show that seeding myoblasts on Matrigel at low confluency results in higher levels of transfection compared to Matrigel negative conditions.

### Up to 93.8% editing was achieved using low confluency and Matrigel-positive conditions

To determine whether CRISPR/Cas9 editing efficiency also improves when cells are seeded on Matrigel-coated wells at low confluency, we transfected all three skMDC lines with guides targeting three separate genes (*MYF5, MYOD1,* and *GREM1*). Each guide should induce a single dsDNA break, which will subsequently be repaired by non-homologous end joining. Both the editing efficiency and mutation type and frequency were calculated automatically by TIDE. The most frequent outcome was a single indel at the target cut site. An example of guide design, PCR, sequencing, and TIDE analyses are shown in Additional file 4.

**Fig 3:**
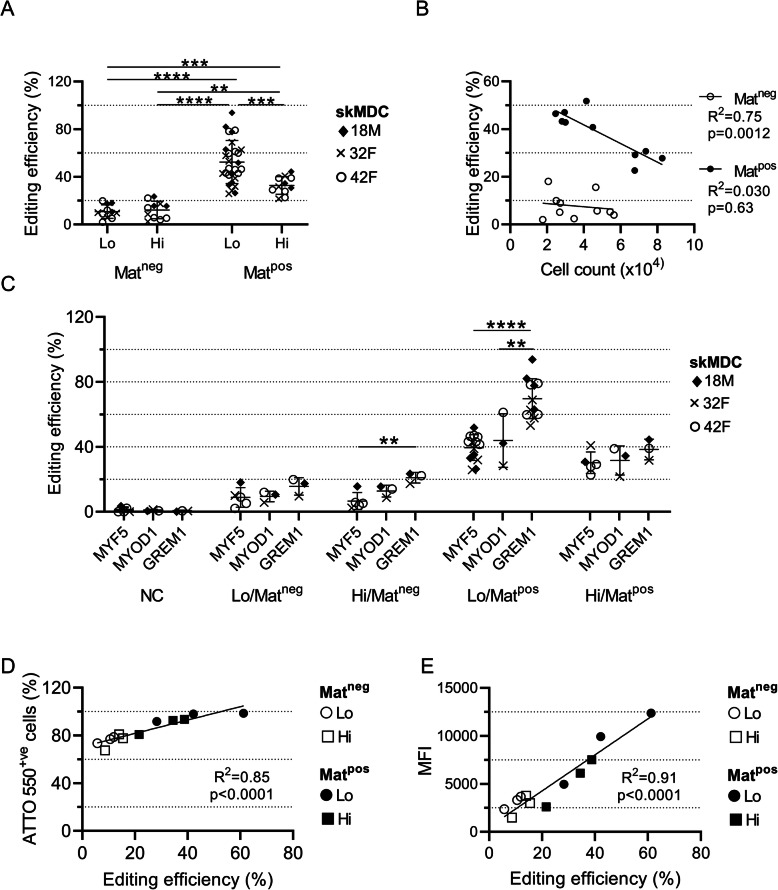
Matrigel and low confluency conditions produced the highest editing efficiency. **A** Compiled results for all editing experiments. Replicates from three skMDC lines were transfected with one of three guides under different conditions. **B** Correlation between editing efficiency and the number of cells at the time of transfection. There is a clear negative relationship between cell count and editing efficiency under Mat^pos^ conditions (*R*^2^ = 0.75; *p* = 0.0012). *n per condition = 5 (1× 18M, 1× 32F, 3x 42F)*. **C** Comparison of editing efficiency between guides within each condition. Different skMDC donor cell lines are indicated with different symbols. Experiments using Lo/Mat^pos^ conditions were repeated to confirm our findings. **D** Correlation between the percentage of ATTO 550^+^ cells and *MYOD1* editing efficiency in three skMDC lines transfected under different conditions. **E** Correlation between MFI and *MYOD1* editing efficiency in three skMDC lines transfected under different conditions. All data points represent individual replicates. Error bars show mean ± SD. ** = p < 0.01; *** = p < 0.001; **** = p < 0.0001. NC = negative control; Lo = low confluency, Hi = high confluency; Mat^*neg*^ *= uncoated wells; Mat*^*pos*^ *= Matrigel coated wells; MFI = median fluorescence intensity*

For all three targets in each skMDC line, the highest editing efficiency was achieved in myoblasts that were transfected at Lo/Mat^pos^ (52.4 ± 17.8%; *n* ≥ 11 per condition), achieving a maximum of 93.8% editing (Fig. 3A). This was significantly higher than the next best set of conditions, Hi/Mat^pos^ (32.8 ± 7.1%, *p* = 0.0007), as well as Hi/Mat^neg^: (12.2 ± 7.0%, *p* < 0.0001) and Lo/Mat^neg^ (10.8 ± 5.4%, *p* < 0.0001). The same pattern was seen when comparing individual targets, although the absolute editing efficiency differed between guides (Fig. 3C). The average editing efficiency of *GREM1* across all conditions was significantly higher than both *MYF5* (*p* = 0.0002) and *MYOD1* (*p* = 0.0012). This difference was most pronounced under Lo/Mat^pos^ conditions, where the average editing efficiency of *GREM1* was 69.65 ± 11.79%, compared to 39.48 ± 7.66% for *MYF5* (*p* < 0.0001) and 43.90 ± 13.49% for *MYOD1* (*p* = 0.0029). While confluency did not impact editing in Mat^neg^ cells (*R*^2^ = 0.030, *p* = 0.63), there was a significant negative correlation between the number of cells at the time of transfection and editing efficiency under Mat^pos^ conditions (*R*^2^ = 0.75, *p* = 0.0012; Fig. 3B). Overall, these data showed that transfecting myoblasts seeded on Matrigel at low confluency produced substantially higher amounts of editing compared to the other conditions.

### The nature of the edits were similar across cell lines, transfection conditions and editing efficiencies

**Fig. 4:**
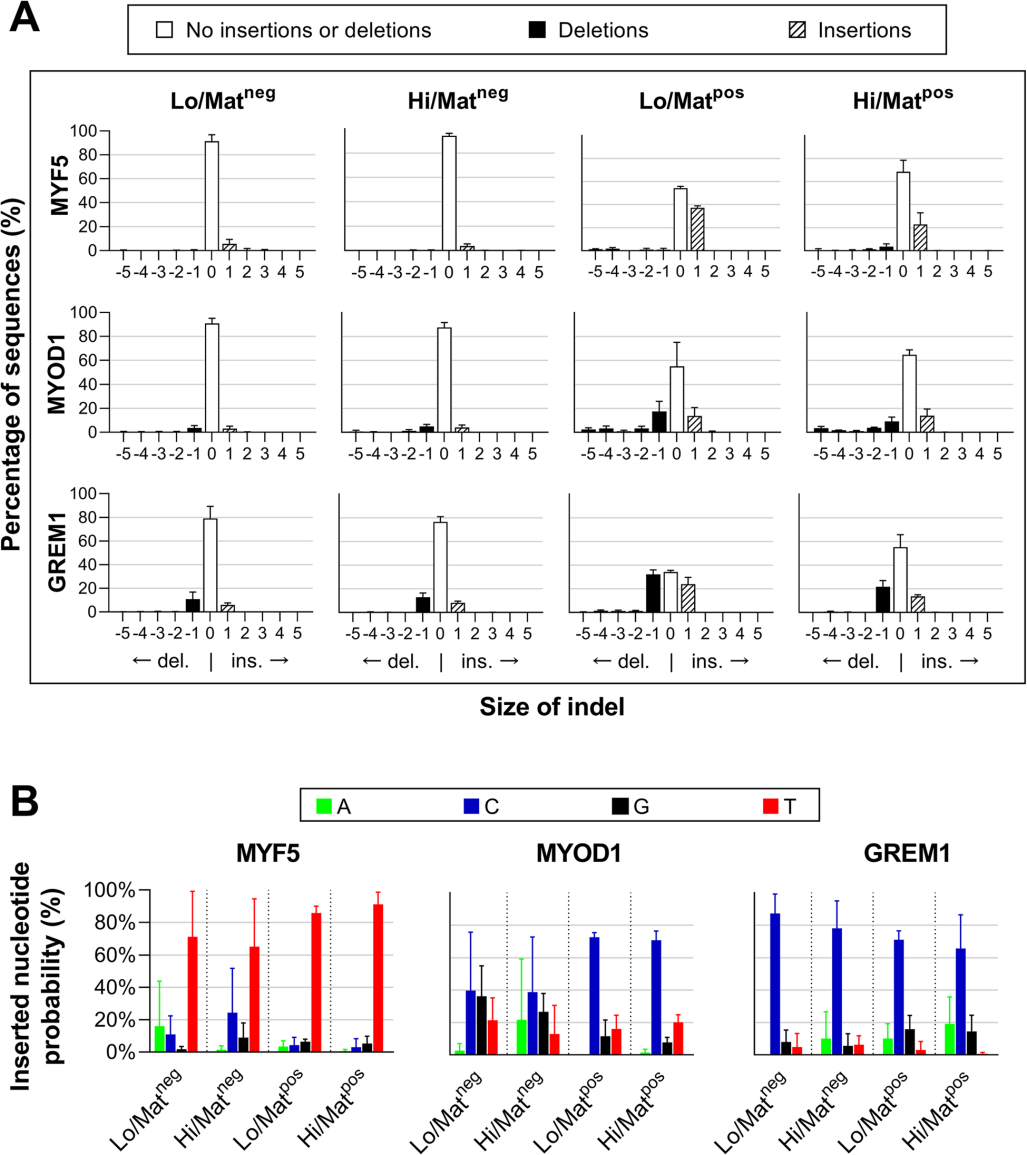
The nature of the edits are similar across cell lines, transfection conditions and editing efficiencies. **A** While the percentage of sequences showing some form of indel is markedly higher in cells transfected under Lo/Mat^pos^ conditions, the proportion and types of indels that occur are similar across the board *(n = 3 per condition – 1× 18M, 1× 32F, 1× 42F)*. **B** The single nucleotide most frequently inserted follow a similar pattern between cell lines and conditions for each target. There is greater variability in the Matrigel negative conditions due to the lower levels of editing *(n = 3 per condition – 1× 18M, 1× 32F, 1× 42F)*

We used data generated by the TIDE algorithm to assess the nature of the edits in the skMDCs. TIDE can identify differently sized indels (default parameter < 10 bp), and, in the cases of single insertions, calculate the frequency of each of the four possible inserted bases (A,G,C,T) [56]. To this end, we investigated the nature of the edits that occurred in each target gene. Regardless of cell line, editing efficiency and transfection conditions, the nature of the indels remained similar for each guide (Fig. 4A). Single nucleotide insertions and/or deletions were most common, accounting for 83.95 ± 17.43% of edits seen. The majority of *MYF5* edited cells contained a single nucleotide insertion, accounting for 82.05 ± 3.62% of identified indels, and was most frequently a thymine (c.249_250insT; Fig. 4B). A single deletion and/or insertion accounted for 74.34 ± 3.08% and 95.37 ± 2.03% of *MYOD1* and *GREM1* edited cells, respectively. The most frequently inserted base in both cases was a single cytosine (*MYOD1*: c.426_427insC; *GREM1*: c.312_313insC). The non-random nature of these indels are in line with previous studies [58–62]. All of these edits are predicted to cause a frameshift early in the transcript, and therefore should result in knockdown of gene expression by nonsense mediated decay.

### Editing efficiency differences are not explained by transfection efficiency differences alone

CRISPR-based editing requires successful delivery of the CRISPR/Cas9 components; however, there is not always a direct correlation between editing efficiency and transfection efficiency [63]. We assessed this relationship in myoblasts edited with fluorescently-labeled (ATTO 550^+^) guides targeting a known muscle gene (*MYOD1*), using FACS to quantify transfection efficiency. Transfection efficiency was positively correlated with editing across the three cell lines under all conditions (*R*^2^ = 0.85, *p* < 0.0001; Fig. 3D); however, the degree of editing was disproportionately higher than could be explained by transfection efficiency alone. For example, there were 1.26 (± 0.01) fold more ATTO 550^+^ cells in Lo/Mat^pos^ conditions compared to Lo/Mat^neg^, but the proportion of edited Lo/Mat^pos^ cells was 4.67 ± 0.50 times higher. These results imply that editing efficiency is influenced by factors other than transfection efficiency.

### Targeting *MYF5* with CRISPR/Cas9 results in decreased levels of *MYF5* and its downstream partner *MYOD1*

**Fig. 5:**
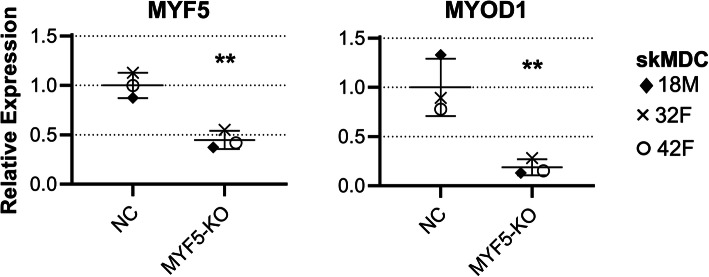
Expression of *MYF5* and *MYOD1* are both decreased in *MYF5* edited cells. Levels of *MYF5* and *MYOD1* in *MYF5*-edited cells (*MYF5-*KO) are shown relative to the average expression of the negative controls (NC). There is a clear decrease in expression of both genes when compared to cells transfected with the scrambled NC guide (n = 3 per condition – 1× 18M, 1× 32F, 1× 42F). Error bars show mean ± SD. ** = p < 0.01

*MYF5* is known to regulate *MYOD1* expression [21, 64–67]. A previous study has shown that silencing *MYF5* expression results in decreased levels of *MYOD1* [21]. To confirm the efficacy and relevance of CRISPR/Cas9 editing in our cells, we measured *MYF5* and *MYOD1* transcript levels in *MYF5* knockout lines (Fig. 5; *n* = 3; 1*×* 18M, 1*×* 32F, 1*×* 42F). The levels of *MYF5* in knockout samples were significantly lower than that of the negative controls (*p* = 0.0023), as was *MYOD1* (*p* = 0.0097; unpaired *t* test).

## Discussion

This study aimed to address the lack of efficient protocols for CRISPR/Cas9-mediated gene editing of human primary myoblasts. The basis of our work was that cell proliferation is positively correlated with transfection efficiency and thus likely to improve editing efficiency. We assessed how different combinations of cell confluency and extracellular matrix membrane (Matrigel) impacted transfection and editing efficiency in primary human myoblasts. We found that skMDCs are most effectively transfected and edited when seeded on Matrigel at low confluency (~ 40%; Fig. 3C). Using these conditions, we edited on average 52.4 ± 17.8% skMDCs within a given population, without the use of cell-sorting or selection. In one instance, an editing efficiency of 93.8% was observed. Editing under Lo/Mat^pos^ conditions was on average >4-fold higher than most protocols currently provided by commercial suppliers (Hi/Mat^neg^), which recommend transfection at high confluencies (60%-90%) and provide no mention of the use of basement membrane matrices. In fact, for all three guides, the lowest editing efficiency attained under Lo/Mat^pos^ conditions was still better than the highest editing efficiency achieved using Hi/Mat^neg^ conditions. While we tested a single guide per target in this study, it is well established that editing activity of different gRNAs varies [68, 69]. Thus, outcomes could be further improved by testing multiple guides per target. Regardless, the activity of poorly performing gRNAs is still improved by the use of our methods. This could be particularly helpful in studies where the position of the gRNA is constrained (e.g., allele-specific deletions, functional assays of disease-causing variants). Overall, this technique has multiple downstream applications both clinically (e.g., genetic therapies) and in research (e.g., disease gene characterization).

Our results unanimously show that of the four conditions tested, the best editing is achieved when transfecting cells that have been seeded on Matrigel at low confluency. This could be due to a dose-effect response, where a larger ratio of RNP complex to each cell increases the likelihood of one or more RNP complexes entering a cell. However, preliminary experiments optimizing the cell:RNP ratio on myoblasts under Lo/Mat^pos^ conditions showed no significant difference in editing efficiency. Increasing concentrations of CRISPR/Cas9 were also associated with higher levels of cell death. While these experiments were not performed on high density cultures, myoblasts are recommended to be kept below 50–60% confluency to prevent spontaneous differentiation. Thus, transfecting at low confluence with low CRISPR/Cas9 concentration simultaneously promotes optimal growth conditions whilst minimizing post-transfection toxicity.

Effective gene editing requires the Cas9 RNP complex to enter the nucleus. The nuclear envelope is largely impermeable to RNP complexes, and thus presents a major barrier to efficient editing. However, during cell division the nuclear envelope is rapidly disassembled before reforming in the daughter cells [63–65]. During this process nearby transfection complexes can become incorporated within the reformed nucleus, where it is then able to access and manipulate the genome [66–70]. Thus, RNPs delivered to cells transfected under conditions that best promote proliferation – i.e., Lo/Mat^pos^—are likely to increase the delivery of intranuclear RNPs, and consequently increase the likelihood of successful editing.

There are several limitations to this study. Transfection efficiency was quantified based solely on the presence of tracrRNA, and does not guarantee that all other components have also been delivered. Thus, some cells may have been transfected with any remaining unbound tracrRNA rather than the entire Cas9-RNP complex, which would bias measures of RNP transfection efficiency. In addition, the proportion of edited to non-edited cells are likely to decrease with time, as unedited cells better retain their ability to proliferate [36]. Further, preliminary studies show reduced differentiation potential in skMDCs transfected with CRISPRMAX reagents, even in the absence of an RNP complex. This may limit the use of this process to the investigation of myoblasts alone. Nevertheless, we believe this remains a viable approach for investigating the effects of gene editing on myoblasts. This is supported by our qPCR results (Fig. 5), which show the expected downstream effects of *MYF5* knockdown on *MYOD1* expression, as has been shown previously [21]. While protein expression studies would have provided further insight into the final outcome of editing, the low endogenous expression of *MYF5* and *MYOD1* (those targets with validated antibodies) made them ill-suited for accurate quantification of changes to protein abundance. This is further complicated by the repression of transcriptional and translational machinery in response to transfection-induced stress [70–75]. Consequently, proteins at low abundance in untreated samples are further reduced in those transfected with scrambled negative control guides (NC), making it difficult to discern a difference in protein levels between NC and edited cells. Preliminary western blot studies using validated antibodies were not sensitive enough for robust detection of MYF5 nor MYOD1 in NC samples, even when the experiment was scaled up over 5-fold (data not shown). While protein analysis is not well-suited for the targets analyzed in this manuscript, this gene editing method is nevertheless applicable to many highly expressed genes that may be better suited to protein analyses.

The benefits gained by using primary cells as a biological model can be easily outweighed by the difficulties inherent in working with them. This is reflected by the predominance of resources and reagents optimized for immortalized rather than primary cell lines. For example, of the 154 cell-specific transfection protocols developed by Invitrogen™ only six are designed for primary cells, and of these six, only one is for human cells [76]. None are tailored specifically for primary myoblasts. By improving the efficiency of CRISPR/Cas9-mediated gene editing in skMDCs, we have made primary myoblasts a viable and attractive option for investigating human muscular genetics. Achieving a high proportion of edited cells in a population reduces the need for downstream cell-selection processes, as robust analyses may be possible on the bulk population for certain applications. This would not be suitable for situations that require a homogenous cell population, although a higher editing efficiency would inherently make selection of edited cells more achievable.

Higher levels of editing also increase the likelihood of homozygous edits. Consequently, tools such as single-cell RNA sequencing [77] can be used to characterize the transcriptome of edited cells. While this protocol is highly effective for editing primary myoblasts, we advise caution in the interpretation of downstream analyses. For example, lipid-based complexes are known to alter gene expression patterns in transfected cells, particularly those involved with cellular stress response pathways [78–81]. Further, as Lipofectamine™ CRISPRMAX™ is a relatively new reagent, no studies have yet assessed the degree to which it can alter gene expression profiles. We recommend the use of a scrambled guide as a control to account for cellular changes resulting from the process of transfection itself.

## Conclusions

Our study has identified a simple, effective, and economical technique to maximize CRISPR/Cas9-mediated gene editing efficiency in primary human myoblasts. We have shown that successful editing of primary myoblasts requires conditions contrary to those recommended by most commercial guidelines. The protocol presented herein could prove a valuable tool for understanding the biology and genetics of human skeletal muscle; and has the potential to be adapted for use in other difficult-to-transfect cell types.

## Supplementary Information

**Additional file 1: Table S1.** GuideRNAs (gRNAs). Information on gRNAs, including commercial name, sequence and location.

**Additional file 2: Table S2.** PCR conditions for Sanger sequencing. Includes primer sequences and thermocycling conditions.

**Additional file 3: Table S3.** qPCR conditions. Includes primer sequences and thermocycling conditions.

**Additional file 4: Figure S1.***MYF5* primer design, PCR, sequence traces and trace decomposition. **(A)** Primers for all targets were designed at least 100 bp away from the gRNA, to ensure high quality reads at the predicted cut site. **(B)** PCR of *MYF5* samples (product size = 630 bp). **(C)** Sample traces showing the cut site and the main edit (single T insertion). This is most striking in the Lo/Mat^pos^ sample, where the signal of the inserted T allele (red peak) is almost equal to the wildtype C allele (blue peak). **(D)** Sequence visualization of control (black) and treated (green) samples. The dotted blue line represents the expected cut site. The green peaks show the proportion of nucleotides in the edited sample that differ from the control nucleotide at the same position. The higher the green peaks after the cut site, the more editing is assumed to have occurred.

## Data Availability

All data generated or analyzed during this study are included in this published article and its supplementary files.
