# Supplementary figures and images for: Improved CRISPR/Cas9 gene editing in primary human myoblasts using low confluency cultures on Matrigel
Source: Skelet Muscle. 2021 Sep 22;11:23. doi: 10.1186/s13395-021-00278-1 (PMC8456651; doi:10.1186/s13395-021-00278-1)

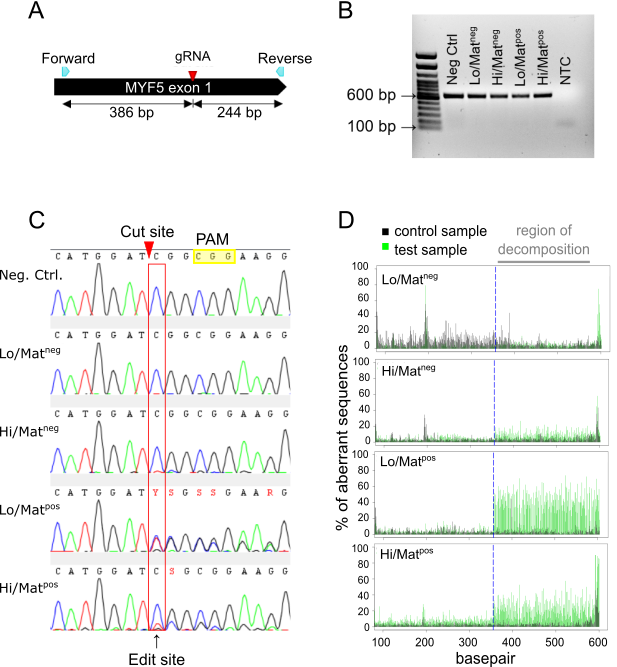

Supplement: Supplementary file 4 — Additional file 4: Figure S1.MYF5 primer design, PCR, sequence traces and trace decomposition. (A) Primers for all targets were designed at least 100 bp away from the gRNA, to ensure high quality reads at the predicted cut site. (B) PCR of MYF5 samples (product size = 630 bp). (C) Sample traces showing the cut site and the main edit (single T insertion). This is most striking in the Lo/Matpos sample, where the signal of the inserted T allele (red peak) is almost equal to the wildtype C allele (blue peak). (D) Sequence visualization of control (black) and treated (green) samples. The dotted blue line represents the expected cut site. The green peaks show the proportion of nucleotides in the edited sample that differ from the control nucleotide at the same position. The higher the green peaks after the cut site, the more editing is assumed to have occurred. [file 13395_2021_278_MOESM4_ESM.png]
